# Interviewing an Elder: Students’ Perceptions of Change in an Introduction to Gerontology Classroom

**DOI:** 10.1093/geroni/igab046.385

**Published:** 2021-12-17

**Authors:** Sarah Hahn

**Affiliations:** Mercy College, Dobbs Ferry, New York, United States

## Abstract

Within the gerontological classroom, high-impact practices and creative assignments have consistently shown to help engage students, optimize learning, and increase positive attitudes toward older adults and aging (e.g., Chonody, 2015; Yamashita, et al., 2018). One such creative assignment, interviewing an older adult, has been cited as both an influential and valuable experience to gerontology students (e.g., O’Hanlon & Brookover, 2002). Although this assignment has been popular in and out of gerontology courses, more data regarding this and student’s understanding is needed. As such, this presentation aims to 1) introduce and establish the value of using the written assignment, "Interview an Elder" in the gerontology classroom and 2) present preliminary qualitative data on how students’ perceptions of older adults changed after the assignment. Using thematic analysis, results suggest that students are not only surprised by what they learned, but have increased positive perceptions of older adults overall.

